# A Giant Sacrococcygeal Teratoma in an Adult Male: A Case Report

**DOI:** 10.7759/cureus.14181

**Published:** 2021-03-29

**Authors:** Bhargav Gajula, Kandhala Srikanth, Farhanul Huda, Harindra Sandhu, Somprakas Basu

**Affiliations:** 1 Surgery, All India Institute of Medical Sciences, Rishikesh, Rishikesh, IND; 2 Surgery, Lala Lajpat Rai Memorial Medical College, Meerut, IND

**Keywords:** sacrococcygeal teratoma, posterior perineal approach, coccygectomy

## Abstract

Sacrococcygeal teratoma (SCT) in adults is very rare with only a few cases reported in the literature. Its presentation in the adult is asymptomatic to a slow-growing cystic tumor with a 1-2% chance for malignant transformation and may attain a huge size causing pressure effect on pelvic and intra-abdominal organs. It can present unusually as a perianal abscess which needs to be evaluated radiologically. We present a giant, long-standing SCT in an adult male patient which presented as a tender fluctuating swelling with spontaneous rupture and whitish discharge in the perianal region masquerading as a perianal abscess. Diagnosis of our case was suspected by clinical examination, ultrasound, and magnetic resonance imaging of the pelvis and histopathology confirmed the diagnosis. It was excised en bloc with coccygectomy and primary wound closure and had a good postoperative recovery. Long-standing perianal swelling in an adult should raise the suspicion of SCT and should be kept in the differentials. The author prefers the posterior perineal approach for excision in Altman type 2, as it has convenient control over the mass during surgery with good cosmetic results as in our case, but the role of coccygectomy to prevent recurrence needs long-term data.

## Introduction

Sacrococcygeal teratoma (SCT) is a rare tumor that presents predominantly in neonates with a prevalence of one in 4000 live births with female preponderance [1]. Congenital SCT can be detected prenatally by ultrasonography. They are postulated to arise from the totipotent somatic cells of the primitive knot [2]. It is composed of multiple tissue types of two or three germ layers.

SCT may present as a cystic mass filled with serous, mucoid, or sebaceous material lined by true epithelium. Patients present with a mass effect on the adjacent organ such as the rectum, urinary bladder, and pelvic vasculature causing the obstruction. Here we report a case of delayed presentation of SCT in an adult sacrococcygeal teratoma presented with perianal swelling with features of rupture. we review the literature to emphasize unusual presentation like perineal abscess as in our case, diagnostic imaging features, and surgical resection modalities.

## Case presentation

A 22-year-old male presented with swelling over the perianal region and lower back with a discharge of whitish material from the swelling.

**Figure 1:**
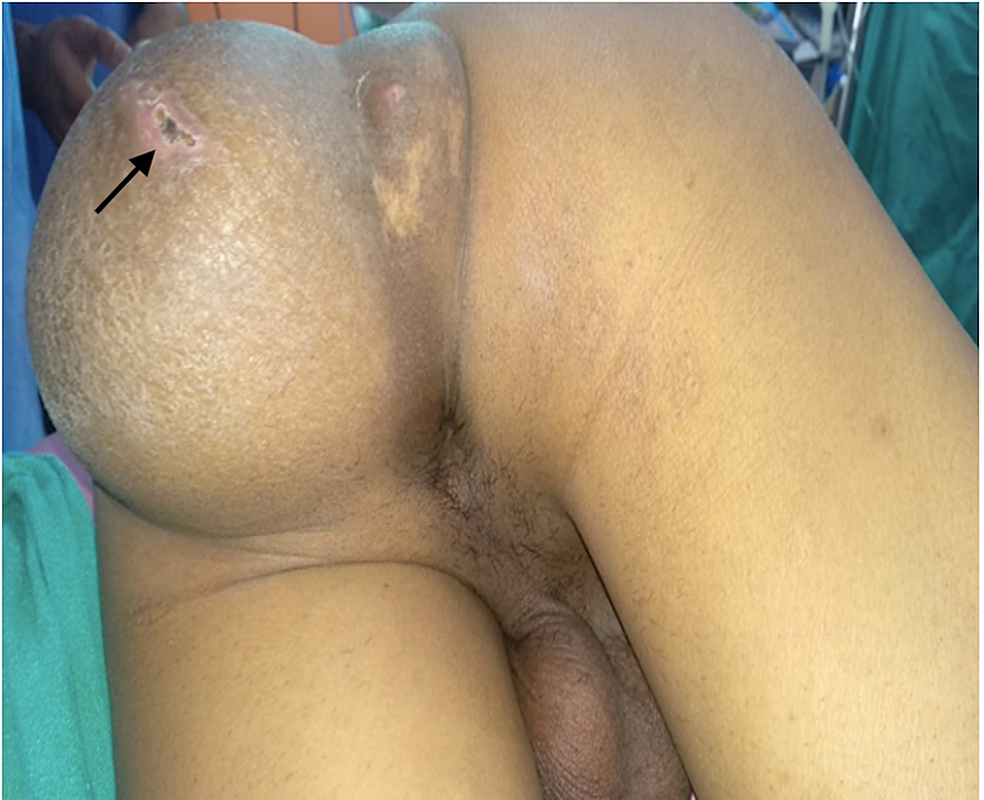
Preoperative prone image showing 12 x 1O cm swelling in the left perianal region and international cleft with a scar at the site of rupture (Black arrow).

**Figure 2:**
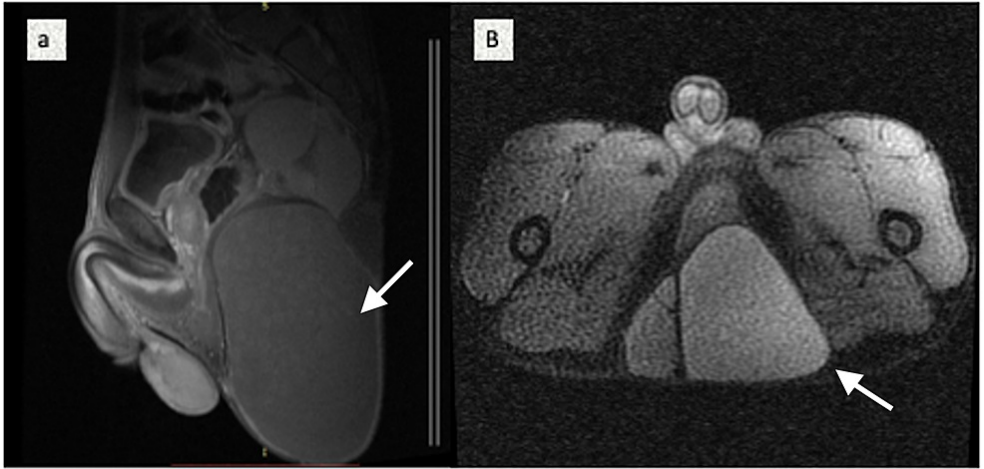
MRI Pelvis A) T1 sagittal section showing cystic lesion of 8x10x20 cm with presacral extension. B) T1 Axial section showing cystic lesion compressing rectum without any obvious extension. MRI: Magnetic Resonance Imaging

The patient had swelling since childhood which increased in size gradually and was associated with dull aching pain and constipation on and off. Local examination reveals 12x10 cm soft cystic tender swelling with fluctuation with no transillumination (Figure 1). Digital rectal examination reveals an extraluminal cystic swelling compressing the rectum from the posterior side and the rectal mucosa was normal. Ultrasound showed a well-defined multiloculated cystic lesion measuring 13x10x10 cm noted involving the left gluteal region showing multiple floating hyperechoic densities and coarse internal echoes. The mass was superiorly extending into to presacral region causing anterior displacement of the rectum and prostate. Magnetic resonance imaging of the pelvis showed a multiloculated cystic lesion in the perianal region externally in the subcutaneous plane in the natal and gluteal folds measuring 8x10x20 cm and superiorly, it is extending into the pelvis into the presacral space till S2 vertebral level (Figure 2).

**Figure 3:**
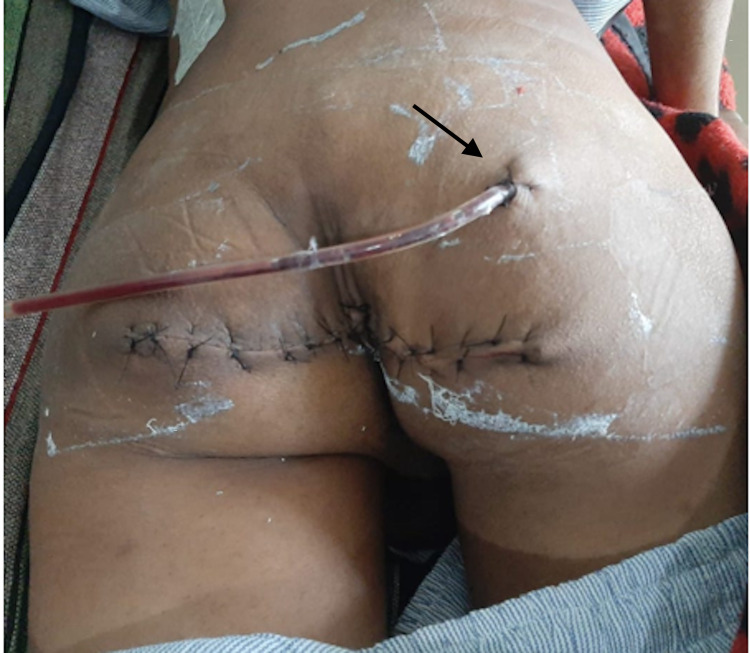
Post-op day 2 image showing primary closure of skin with subcutaneous drain in-situ In right upper quadrant of the gluteal region (black arrow).

The cysts showed central diffusion restriction and smooth peripheral rim enhancement and the diagnosis of SCT Altman Type II was made [3]. Preoperative serum tumor markers like alpha-fetoprotein, carcinoembryonic antigen, human chorionic gonadotropin, lactate dehydrogenase, and cancer antigen 125 (CA-125) were within normal limits. The patient underwent complete surgical excision of mass by posterior approach through an elliptical incision and intraoperatively a cystic mass was found abutting rectum, anal canal, levator ani, and puborectalis muscle. It was carefully resected from the surrounding structures by entering the presacral plane, coccygectomy was not done, the subcutaneous closed suction drain was placed and skin was closed primarily by 3.0 nylon (Figure 3).

**Figure 4:**
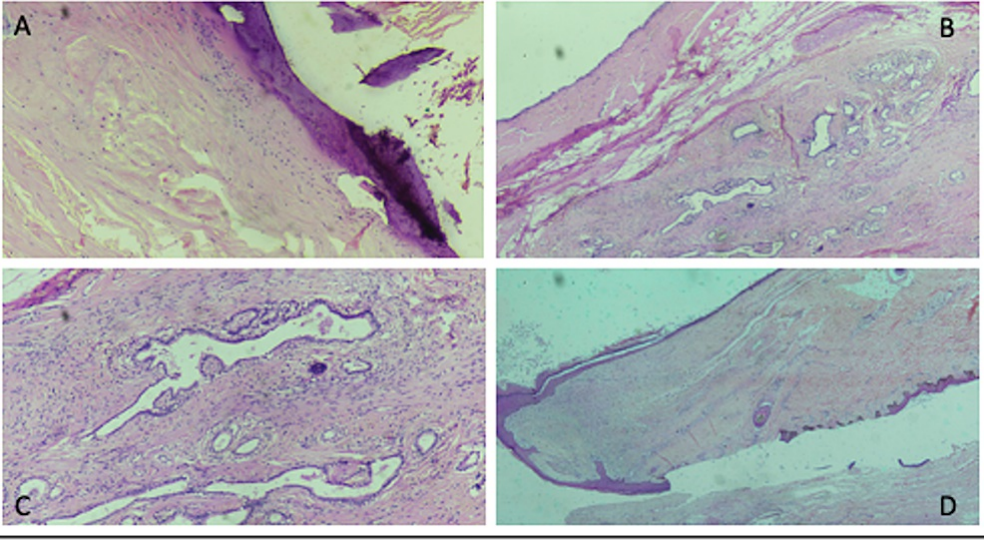
Histopathology H&E Stain A) Bone B) Cuboidal epithelium C) Glandular elements D) Adnexa-hair H&E: Hematoxylin & Eosin

The excised specimen was sent for histopathological examination. The postoperative course of the patient was uneventful and discharged in satisfactory condition. Histopathology of the patient revealed solid and cystic areas with a cystic cavity lined stratified squamous epithelium in some sections and by columnar epithelium at places, numerous entrapped hair follicles and adnexal structures are also seen. The solid areas have shown disorganized muscle bundles, adipocytes, occasional bony, and cartilaginous tissue (Figure 4). The patient was on follow-up every six months and was asymptomatic clinically to date on an 18-month follow-up period.

## Discussion

Teratomas are Germ cell tumors composed of any or all the three germ layer elements which are the most frequent solid congenital tumors in the fetus and newborn in the sacrococcygeal region. However, teratomas have been identified throughout the body.

SCT is rare in adults with a few published case reports and series with a lack of information on its incidence in the general population. Adult SCT tends to occur more frequently in females with a female to male ratio of 4-10:1 [4].

Its presentation in adults is often asymptomatic and it is difficult to diagnose SCT without clinical symptoms and signs and can be found incidentally on physical examination or by chance during imaging studies. The usual presentation includes sacrococcygeal pain, mass, constipation, and urinary disturbances. Sometimes SCT can be associated with infections presenting as ulcers that mimic fistula-in-ano, wherein digital rectal examination (DRE) helps to rule to underlying fistula-in-ano.

Adult SCT is mostly seen as intra-pelvic mass compared to neonatal variety which mostly presents as externally visible mass [5]. Pathologically SCT is categorized as Mature (well-differentiated) benign Teratoma and immature (poorly differentiated) Teratoma with malignant potential. The incidence of malignant transformation of adult teratomas is much lower in contrast to infantile type with an incidence of about 1-2% [4].

Ultrasonography serves as an initial diagnostic tool to assess cystic and solid components but often inconclusive in determining intrapelvic extension. MRI pelvis is the most reliable diagnostic tool in evaluating components of SCT based on signal intensity. But features of malignancy like local invasion and regional lymph node enlargement warrant a computerized tomography (CT) scan for staging along with serum tumor markers of alpha-fetoprotein, carcinoembryonic antigen, human chorionic gonadotropin, lactate dehydrogenase, and CA-125.

Complete surgical excision with coccygectomy is the most acceptable treatment for SCT [6]. However, the excision of the coccyx to prevent recurrence remains controversial [7], which needs long-term follow-up data. Generally, a tumor that extends above the S3 requires an anterior approach by either laparotomy or laparoscopy [8], whereas below S3 are removed by posterior only (para sacral) approach [9]. A combined anterior-posterior approach is used for large tumors [10]. The raw area thus created by excision can be closed primarily or by local flap coverage.

## Conclusions

SCT in adults can be difficult to diagnose because of a lack of typical clinical symptoms and signs. The reason for delayed presentation in adults being unawareness, lack of serious complications despite cosmetic blemish, and the rural background. Radiological examination is beneficial for preoperative diagnosis. Therefore preoperative road map with magnetic resonance imaging (MRI) of the pelvis is necessary for appropriate planning of the optimal surgical procedure and helps in the Altmann classification of SCT. The choice of surgical approach and surgical resection modality depends on the size, location, and components of the tumor. Most adult SCTs are benign; the surgical outcome for the malignant SCT was also good after complete resection, and even after recurrence, the surgical outcome was good after re-resection. But the role of coccygectomy to prevent recurrence needs long-term follow-up data.
